# Area Under the Expiratory Flow–Volume Curve (AEX): Assessing Bronchodilator Responsiveness

**DOI:** 10.1007/s00408-020-00345-2

**Published:** 2020-03-24

**Authors:** Octavian C. Ioachimescu, James K. Stoller

**Affiliations:** 1 Division of Pulmonary, Allergy, Critical Care and Sleep Medicine, School of Medicine, Emory University, Atlanta VA Sleep Medicine Center, 250 N Arcadia Ave, Decatur, GA 30030 USA; 2 Education Institute, Cleveland Clinic, 9500 Euclid Ave, Cleveland, OH USA

**Keywords:** Lung function, Spirometry, Lung volumes, Area under flow–volume curve, Bronchodilator response

## Abstract

**Background:**

Area under expiratory flow–volume curve (AEX) is a useful spirometric tool in stratifying respiratory impairment. The AEX approximations based on isovolumic flows can be used with reasonable accuracy when AEX is unavailable. We assessed here pre- to post-bronchodilator (BD) variability of AEX_4_ as a functional assessment tool for lung disorders.

**Methods:**

The BD response was assessed in 4330 subjects by changes in FEV_1_, FVC, and AEX_4_, which were derived from FVC, peak expiratory flow, and forced expiratory flow at 25%, 50%, and 75% FVC. Newly proposed BD response categories (negative, minimal, mild, moderate and marked) have been investigated in addition to standard criteria.

**Results:**

Using standard BD criteria, 24% of subjects had a positive response. Using the new BD response categories, only 23% of subjects had a negative response; 45% minimal, 18% mild, 9% moderate, and 5% had a marked BD response. Mean percent change of the square root AEX_4_ was 0.3% and 14.3% in the standard BD-negative and BD-positive response groups, respectively. In the new BD response categories of negative, minimal, mild, moderate, and marked, mean percent change of square root AEX_4_ was − 8.2%, 2.9%, 9.2%, 15.0%, and 24.8%, respectively.

**Conclusions:**

Mean pre- to post-BD variability of AEX_4_ was < 6% and stratified well between newly proposed categories of BD response (negative, minimal, mild, moderate and marked). We suggest that AEX_4_ (AEX) could become a useful measurement for stratifying dysfunction in obstructive lung disease and invite further investigation into indications for using bronchodilator agents or disease-modifying, anti-inflammatory therapies.

## Introduction

Interpretation of Pulmonary Function Testing (PFT) relies mainly on comparing measured flows and volumes with predicted reference intervals derived on healthy subjects from similar populations [1–3]. In spirometry, forced vital capacity (FVC), forced expiratory volume in 1 s (FEV_1_), FEV_1_/FVC ratio, and several isovolumic flows represent the main measurements used for defining respiratory impairment. Additionally, testing before and after inhaled bronchodilator (BD) administration has been widely used, especially for diagnosis and therapeutic monitoring in asthma, chronic obstructive pulmonary disease (COPD), various overlap syndromes, and in other conditions. Various definitions of bronchodilator responsiveness have been proposed, reflecting the complexity of defining reversibility of airflow obstruction [4–11]. The 2005 joint ATS/ERS guidelines defined a significant BD response as an absolute 200 mL and a 12% change in either FEV_1_ or FVC [12]. Recently, Hansen et al. [13] suggested re-defining BD reactivity by using only FEV_1_ or percent changes, and by differentiating between negative, minimal, mild, moderate and marked responses by using the following thresholds: ≤ 0 mL/≤ 0%, ≤ 90 mL/≤ 9%, ≤ 160 mL/≤ 16%, ≤ 260 mL/≤ 26%, and > 260 mL/> 26%, respectively. Their study also correlated the new BD response categories with radiological measurements, exercise performance, dyspnea, obstructive lung disease exacerbation frequency and quality of life scores [13].

In previous publications we examined the use of a spirometric parameter, area under expiratory flow–volume curve (AEX) as an alternative metric for diagnosing and stratifying functional impairment [14–16]. Using this measurement also promises to lessen dependency on body plethysmography or other methods employed as the gold standard for functional assessment, which is costly, elaborate and impractical in either point-of-care setting or in large epidemiological studies. The AEX (expressed in L^2^ s^−1^) is the integral function of the variable flow by volume exhaled during a forced respiratory maneuver. While AEX can be easily computed by any modern digital spirometry acquisition system, it is currently offered by only a minority of PFT platform developers. In this context, our earlier studies assessed the utility of several AEX approximations derived from FVC and available instantaneous flows measurements (AEX_1_, AEX_2_, AEX_3_ and AEX_4_) [17]. Deriving approximated values of the area under the flow–volume loop from widely available spirometric parameters, both before and after BD administration, may mitigate the impact of AEX unavailability in some PFT programs, thereby extending the applicability of this novel measurement.

The current study assesses the pre- to post-BD variability of these AEX approximations, and correlates the association between these measurements and new, emerging, BD response criteria and categories.

## Methods

The working database included 13,954 consecutive tests performed on distinct adult subjects in the Atlanta Veterans Affairs Medical Center PFT Laboratory between January 1st, 2009 and December 31st, 2015. The analyses were performed on a sub-group of subjects who underwent same-day, valid, and ATS/ERS quality-acceptable pre- and post-BD spirometry (*n* = 4330). The largest values among all pre- and post-BD trials have been selected.

Respiratory function assessments were performed in accord with the current ATS/ERS standards and recommendations [1, 18, 19]. Functional measurements were acquired using a Jaeger MasterLabPro system (Wurzberg, Germany), and the most recent and comprehensive reference values, the Global Lung Initiative (GLI) equations sets, were used for spirometry interpretation [2, 20]. Per ATS/ERS recommendations [12], an obstructive ventilatory defect was defined as FEV_1_/FVC ratio below the lower limit of normal (LLN) and a normal FVC. Restriction was diagnosed when the following three criteria were satisfied: normal FEV_1_/FVC ratio, FVC < FVC_LLN_, and Total Lung Capacity (TLC) < TLC_LLN_. If all three FEV_1_/FVC ratio, FVC and TLC were below their LLNs_,_ then a diagnosis of mixed ventilatory defect was established. In these analyses, small airways disease was not included as a distinct category. Lung volume and DLCO reference values were those of Crapo et al. [21, 22].

As detailed elsewhere [17], we defined four spirometric parameters, AEX_1_ through AEX_4_, which were calculated as the sums of the areas of triangles and trapezoids delineated by pre-specified volumes and the respective isovolumic flows. For example, AEX_4_ was constructed from FVC and the following four flows: PEF, FEF_25_, FEF_50_ and FEF_75_, per the following formula [17]:

$${\text{AEX}}_{{4}} = \, \left[ {{\text{FEV}}_{{{\text{PEF}}}} *{\text{PEF }} + \, \left( {{\text{PEF}} + {\text{FEF}}_{{{25}}} } \right)*\left( {0.{25}*{\text{FVC}} - {\text{FEV}}_{{{\text{PEF}}}} } \right) \, + \, \left( {{\text{FEF}}_{{{25}}} + {\text{FEF}}_{{{5}0}} } \right) \times 0.{25} \times {\text{FVC }} + \, \left( {{\text{FEF}}_{{{5}0}} + {\text{FEF}}_{{{75}}} } \right) \times 0.{25} \times {\text{FVC }} + {\text{ FEF}}_{{{75}}} \times 0.{25} \times {\text{FVC}}} \right]/{2}$$

Similarly, AEX_1–3_ were derived as follows [17]:

$${\text{AEX}}_{{1}} = \, \left( {{\text{PEF}} \times {\text{FVC}}} \right)/{2}$$

$${\text{AEX}}_{{2}} = \, \left[ {{\text{FEV}}_{{{\text{PEF}}}} \times {\text{PEF }} + \, \left( {{\text{PEF}} + {\text{FEF}}_{{{5}0}} } \right) \times \left( {0.{5} \times {\text{FVC}} - {\text{FEV}}_{{{\text{PEF}}}} } \right) \, + {\text{ FEF5}}0 \times 0.{5} \times {\text{FVC}}} \right]/{2}$$

$${\text{AEX}}_{{3}} = \, \left[ {{\text{FEV}}_{{{\text{PEF}}}} *{\text{PEF }} + \, \left( {{\text{PEF}} + {\text{FEF}}_{{{25}}} } \right) \times \left( {0.{25} \times {\text{FVC}} - {\text{FEV}}_{{{\text{PEF}}}} } \right) \, + \, \left( {{\text{FEF}}_{{{25}}} + {\text{FEF}}_{{{75}}} } \right) \times 0.{5} \times {\text{FVC }} + {\text{ FEF}}_{{{75}}} \times 0.{25} \times {\text{FVC}}} \right]/{2}$$

For clarity, we limit the data presented in this article to AEX_4_, although the analyses pertaining to AEX_1_, AEX_2_ and AEX_3_ showed similar results, albeit with smaller coefficients of variation (mainly due to lower intrinsic variability of PEF, FEF_25_ and FEF_50_).

Descriptive analyses of the available variables were performed. Categorical variables were presented as frequencies or percentages. Continuous variables were described as means ± standard deviation (SD, for normally distributed variables) or as medians and 25th–75th interquartile ranges (IQR, for non-normal distributions). Student’s t test and analysis of variance were used to compare mean values, while categorical variables were compared using χ^2^ (likelihood ratio) test. The Tukey–Kramer HSD method was used to compare means among pairs when the variances were similar, while Wilcoxon or Kruskal–Wallis rank sum tests were performed as non-parametric methods when variances were unequal, as appropriate.

Statistical significance was defined a priori as *p* < 0.001. Analyses were performed using JMP Pro 15 statistical software (SAS Institute, Cary, NC, USA).

Institutional research approvals were obtained to conduct the study (Cleveland Clinic IRB EX#0504/EX#19-1129 and Emory University IRB 00049576).

## Results

During the inclusion period, 4330 subjects underwent same-day, acceptable pre- and post-BD spirometry testing. Racial profiles were similar to the larger database of all subjects tested in the PFT Laboratory: 2183 (51%) were self-identified as Black or African American; 2044 (48%) were White or Caucasian; < 2% were Hispanic or Latino. Eleven per cent (*n* = 494) were women and 89% (*n* = 3836) were men. Age characteristics were also similar to the base cohort: 59 ± 12 [mean ± SD] and 60 (51–66) [median (IQR)] years. Median (IQR) height, weight and body mass index or BMI were 1.78 (1.73–1.83) m, 91 (79–107) kg, and 29 (25–33) kg/m^2^, respectively. Based on the standard diagnostic criteria, 30%, 57%, 9% and 5% were diagnosed with normal pattern, obstruction, restriction or mixed ventilatory defect, respectively.

During the test day and before albuterol administration, 77% of the subjects had additional lung volume determinations by body plethysmography, 7% by the helium dilution method and approximately 83% underwent DLCO measurements. Pre-BD TLC, Inspiratory Capacity (IC), IC/TLC, and DLCO are shown in Table 1. Approximately 9% of the subjects had a baseline IC/TLC less than 0.25.

**Table 1:** Functional measurements before and after bronchodilator (BD) in the test sub-group (*n* = 4330)

Parameter	Mean pre-BD	Mean post-BD	Delta	95% CI	Mean % change
TLC—mean ± SD (L), % predicted	6.4 ± 2.1, 94.2%				
IC—mean ± SD (L), % predicted	2.5 ± 0.7, 68.0%				
IC/TLC—mean ± SD, % predicted	0.40 ± 0.12, 68.4%				
DLCO—mean ± SD (mL/min/mmHg), % predicted	17.4 ± 6.6, 64.5%				
PEF (L)	5.996	5.952	− 0.044	− 0.078 to − 0.010	1.2
FET (s)	10.961	10.647	− 0.134	− 0.396 to − 0.232	1.5
FET_PEF_ (s)	0.149	0.157	0.008	0.006 to 0.011	1.2
FIVC (L)	3.091	3.194	0.103	0.086 to 0.120	2.1
FIV_1_ (L)	2.332	2.371	0.039	0.017 to 0.061	1.0
FEV_0.5_ (L)	1.766	1.867	0.100	0.093 to 0.108	7.3
FEV_1_ (L)	2.334	2.465	0.134	0.123 to 0.138	6.6
FEV_2_ (L)	2.715	2.854	0.139	0.131 to 0.146	5.9
FEV_3_ (L)	2.950	3.093	0.143	0.136 to 0.151	5.6
FEV_6_ (L)	3.281	3.417	0.136	0.128 to 0.144	4.7
FVC (L)	3.528	3.639	0.110	0.101 to 0.119	3.6
FEV_1_/FVC	0.662	0.680	0.0182	0.0167 to 0.0198	2.2
FEV_1_/FEV_6_	0.705	0.718	0.013	0.012 to 0.014	1.9
FEF_25_ (L s^−1^)	4.622	4.806	0.184	0.146 to 0.221	10.8
FEF_50_ (L s^−1^)	2.552	2.863	0.310	0.286 to 0.335	20.0
FEF_75_ (L s^−1^)	0.645	0.784	0.140	0.130 to 0.150	29.8
FEF_25-75_ (L s^−1^)	1.662	1.915	0.253	0.238 to 0.268	20.4
AEX_1_ (L^2^ s^−1^)	11.100	11.344	0.243	0.167 to 0.320	− 1.0
AEX_2_ (L^2^ s^−1^)	9.693	10.413	0.721	0.652 to 0.789	4.4
AEX_3_ (L^2^ s^−1^)	9.047	9.544	0.497	0.435 to 0.558	3.2
AEX_4_ (L^2^ s^−1^)	8.966	9.726	0.760	0.697 to 0.823	5.9
Sqrt AEX_1_ (L min^−0.5^)	3.247	3.211	0.036	0.025 to 0.047	0.3
Sqrt AEX_2_ (L min^−0.5^)	2.975	3.087	0.112	0.102 to 0.122	3.0
Sqrt AEX_3_ (L min^−0.5^)	2.869	2.951	0.082	0.072 to 0.091	2.3
Sqrt AEX_4_ (L min^−0.5^)	1.665	1.702	0.037	0.034 to 0.040	3.7

**Fig. 1:**
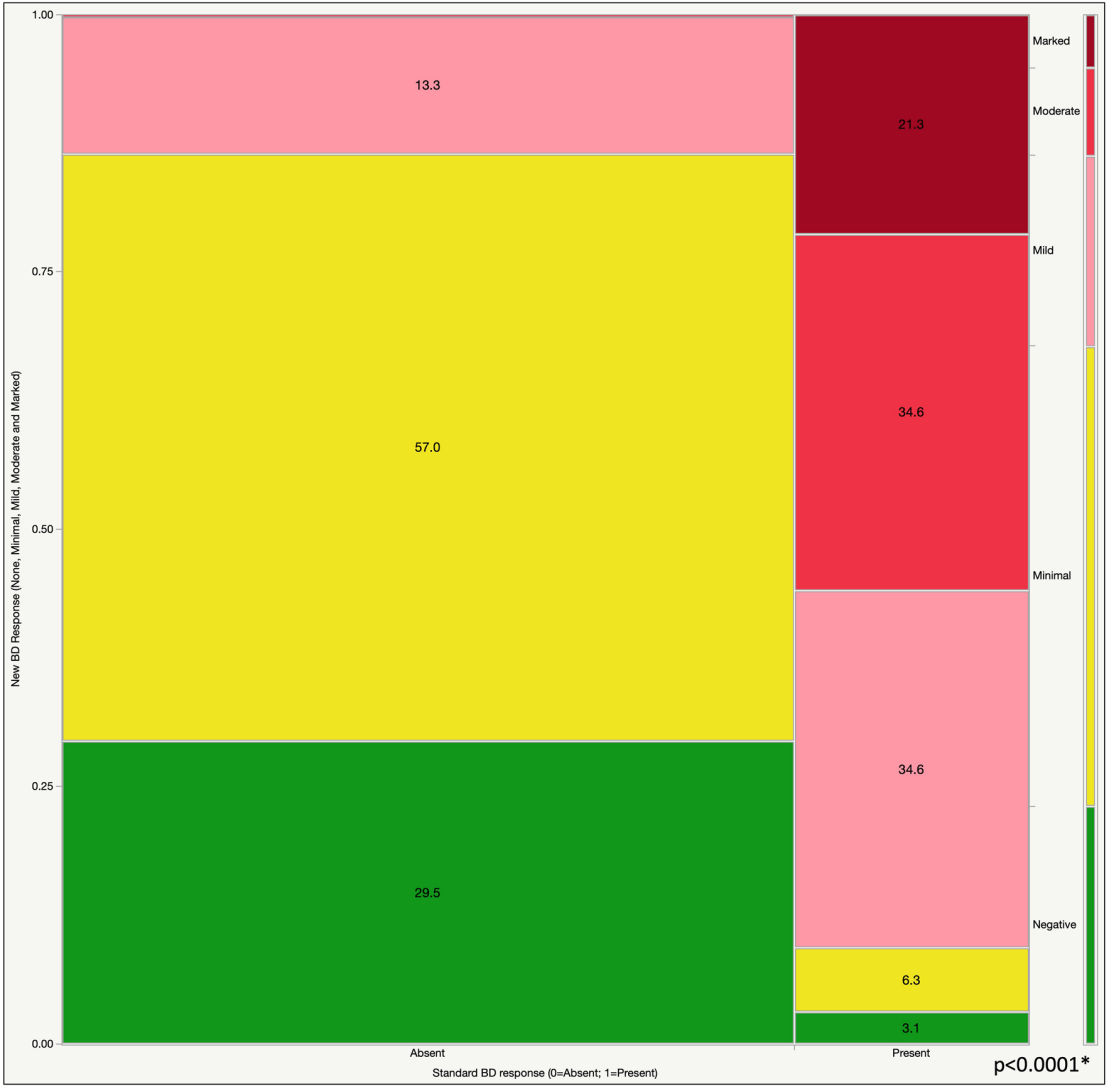
Mosaic plot showing a contingency analysis of new BD response (none, minimal, mild, moderate and marked) by standard BD response (0 = absent; 1 = present)

Using the standard ATS/ERS bronchoresponsiveness criteria (i.e., 12% and 200 ml in FEV_1_ or FVC), 24% of the tested subjects had a positive response. Using the new BD response categories proposed by Hansen et al. [13], only 23% of subjects had a negative response; 45% had minimal, 18% mild, 9% moderate and 5% had a marked BD response. Figure 1 shows the mosaic plot of the newer vs. the standard BD response categories.

Notably, 3.1% of the subjects categorized as having a significant BD response by standard criteria were re-classified as a negative BD response by the new definitions. Alternatively, only 29.5% of those considered by standard criteria to be without a significant BD response maintained a negative BD response by the new categorization.

Table 1 shows the main spirometric parameters of the test set before (pre-BD) and after 2 puffs (400 mcg) of inhaled albuterol (post-BD). The largest pre- vs post-BD variability was noted in the isovolumic flows FEF_25_, FEF_50_, FEF_75_ and FEF_25–75_, i.e., 10.8%, 20.0%, 29.8% and 20.4%, respectively. Despite the fact that AEX_2_, AEX_3_ and AEX_4_ are computed based on the above flows, their percent pre- to post-BD changes were small overall (< 6%, Table 1). Mean sqrt AEX_4_% change was higher in Whites vs Blacks (4.6% vs 2.9%, *p* < 0.0001) and in men vs women (3.9% vs 2.4%, *p* = 0.0091). Weight, height, BMI, body surface area and age were not significant covariates. Interestingly, mean sqrt AEX_4_% change was 0.1%, 0.2%, 5.2%, 5.7% and 27.8% in restriction, normal pattern, mixed ventilatory defects, obstruction and small airway disease, respectively.

**Fig. 2:**
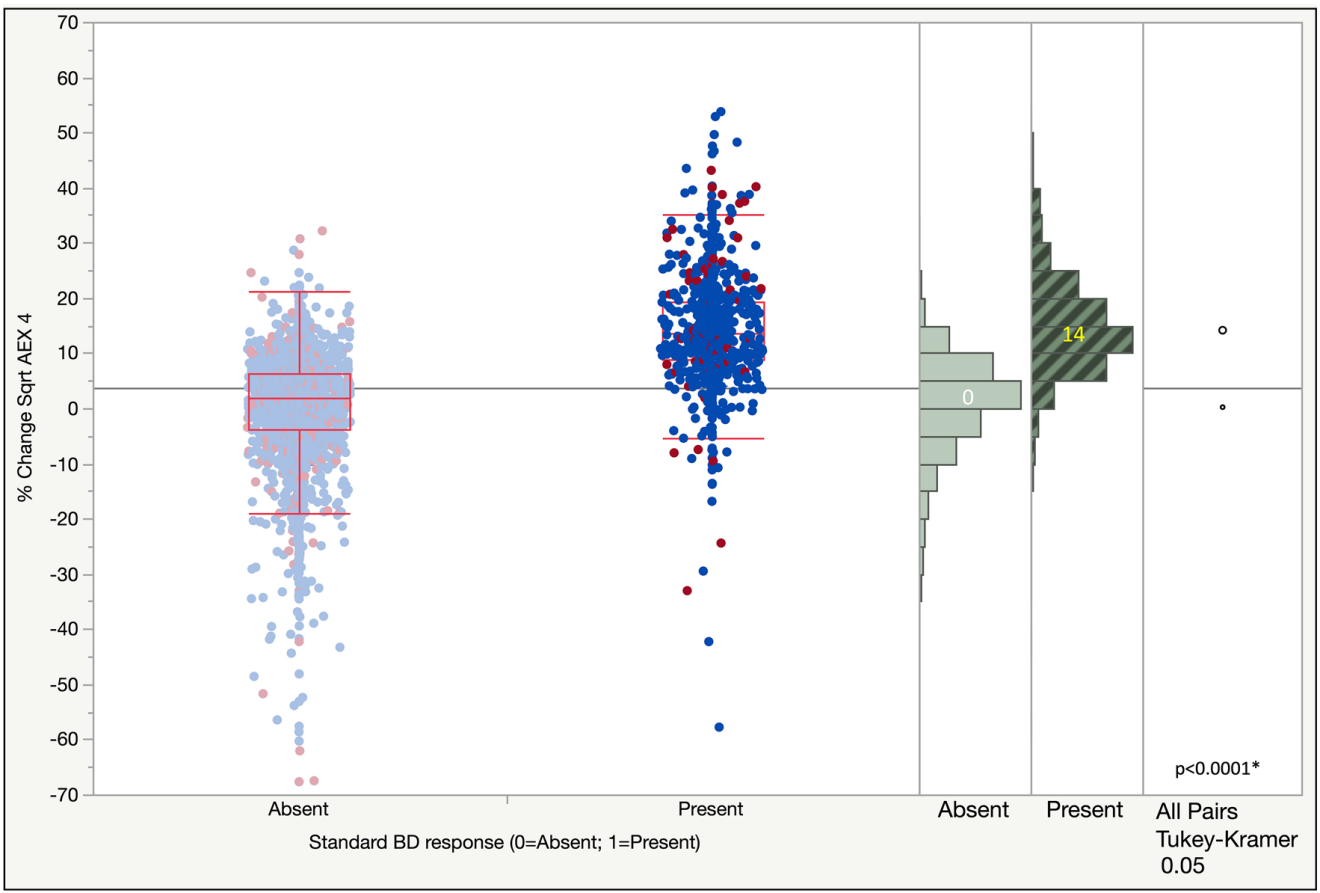
One-way analysis of % change of Sqrt AEX_4_ by standard Bronchodilator (BD) response (absent vs present). Blue: men; red: women; dark color (selected): Positive bronchodilator response by standard BD response criteria

**Fig. 3:**
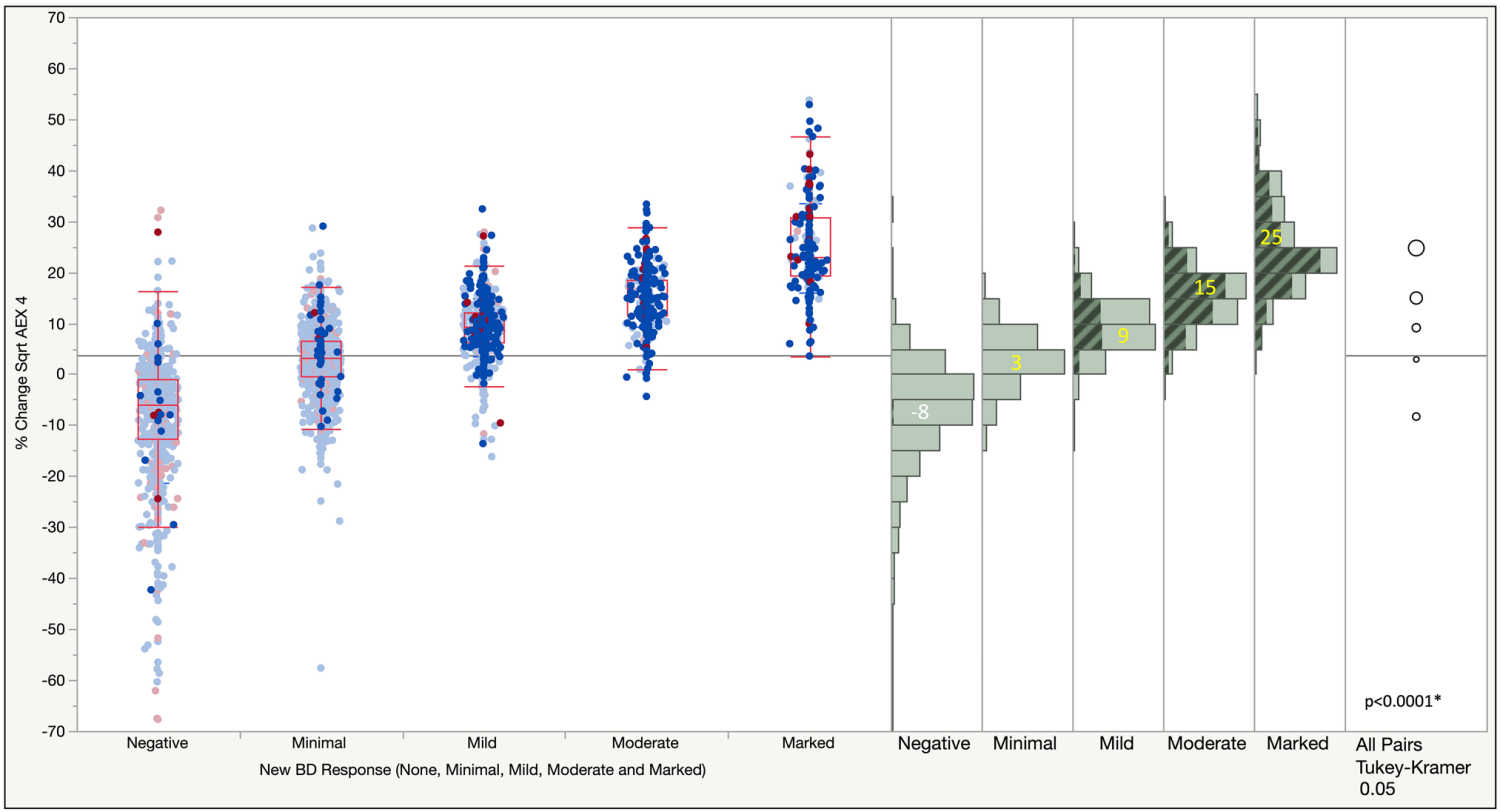
One-way analysis of % change of Sqrt AEX_4_ by New bronchodilator (BD) response (none, minimal, mild, moderate or marked by FEV_1_ absolute *or* percent changes ≤ 0 mL/≤ 0%, ≤ 90 mL/≤ 9%, ≤ 160 mL/≤ 16%, ≤ 260 mL/≤ 26%, and > 260 mL/> 26%, , respectively). Blue: men; red: women. Dark color (highlighted): positive response by standard BD response criteria

Mean percent change of the sqrt AEX_4_ was 0.3% and 14.3% in the negative and positive standard BD response groups, respectively (Fig. 2). In the new BD response categories of negative, minimal, mild, moderate and marked, mean % change of sqrt AEX_4_ was − 8.2, 2.9, 9.2, 15.0 and 24.8%, respectively (Fig. 3).

**Fig. 4:**
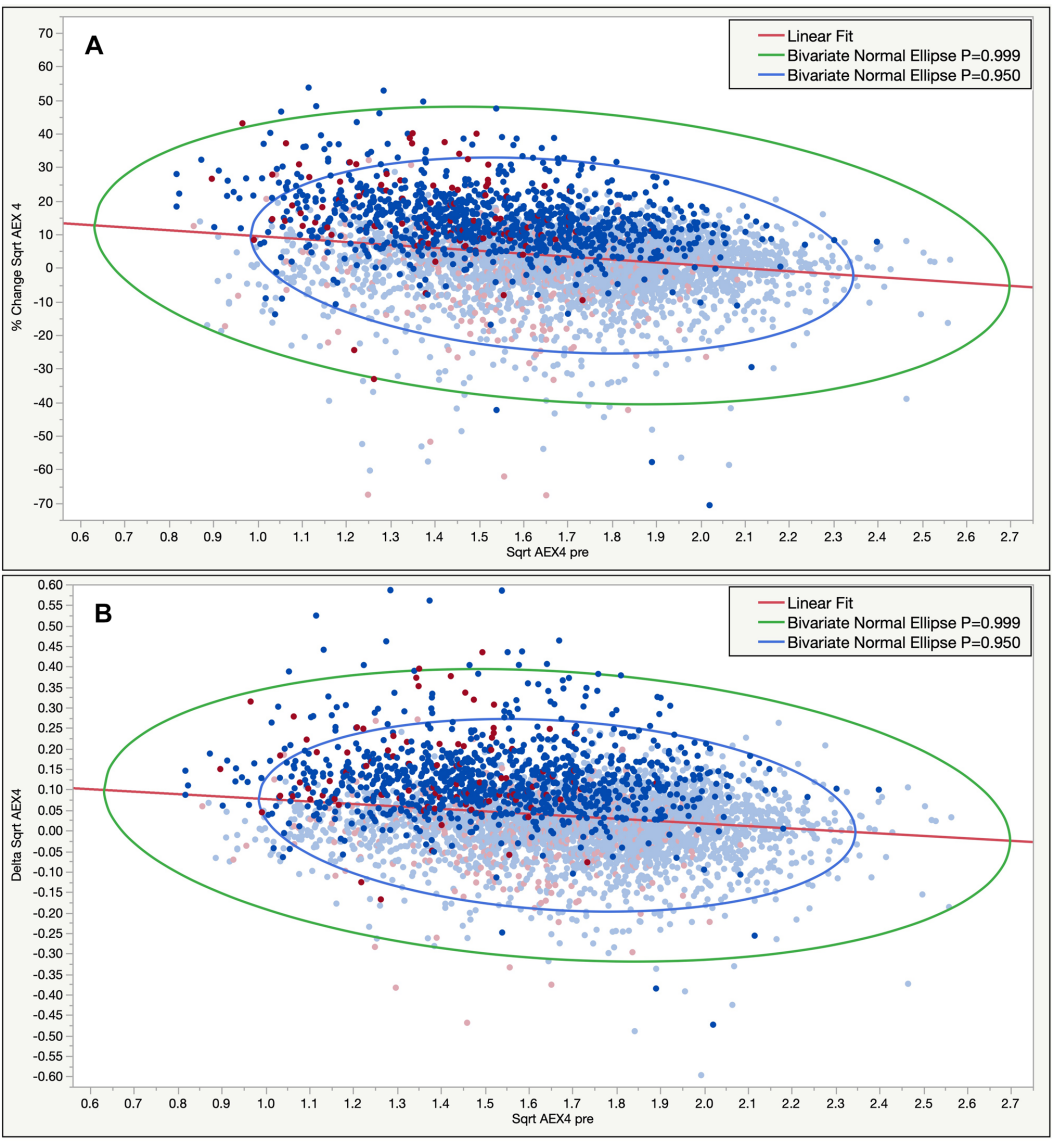
Percent change in Sqrt AEX_4_ (**a**) and Delta Sqrt AEX_4_ post–pre BD (**b**) by AEX_4_ pre-BD. Blue: men; red: women. Dark color (highlighted): positive response by standard BD response criteria

Given that FEV_1_ absolute and % changes tend to move in opposite direction, especially at extremes, we also analyzed the performance of sqrt AEX_4_% and absolute changes versus baseline (pre-BD) sqrt AEX_4_ values (Fig. 4). As noted, despite a descending trend towards higher baseline AEX_4_ (on X axis), the dependency was much lower than for the traditional measurements such as FEV_1_ and FVC.

**Fig. 5:**
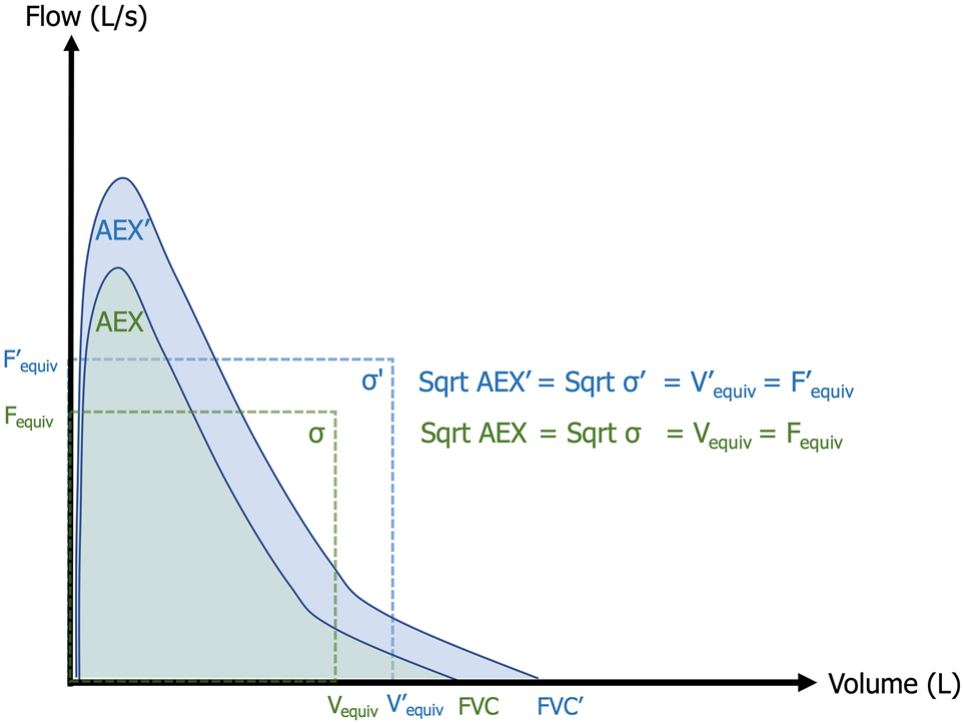
AEX—the integral function of flow by volume during a forced exhalation maneuver. The dotted areas delineate the AEX-equivalent squares (equal scalar sides, i.e., *V*_equiv_ = *F*_equiv_ = Sqrt σ = Sqrt AEX). Green: pre-bronchodilator; Blue : post-bronchodilator. *AEX* area under expiratory flow–volume curve, *FVC* forced vital capacity, *σ* sigma (square area), *Sqrt* square root transformation ( )

To explain the significance of the sqrt AEX (or sqrt AEX_4_), we illustrate in Fig. 5 the AEX-equivalent area square, which introduces two novel physiological concepts: equivalent Flow (*F*_equiv_) and Volume (*V*_equiv_)_,_ i.e., the flow and volume that have the following scalar relationship: Sqrt AEX = *V*_equiv_ = *F*_equiv_. The *V*_equiv_ is highly correlated with FEV_0.5_ (linear fit *R*^2^ = 0.98), FEV_1_ (*R*^2^ = 0.96) and FEV_2_ (*R*^2^ = 0.89), perhaps a reflection of the fact that this portion of the flow–volume curve is generated during the first second of the forced exhalation, and that FEV_1_ and FEV_0.5_ are in close proximity, overriding the *V*_equiv_ (Fig. 5).

## Discussion

The main findings in this analysis are that the pre- to post-BD variability of AEX_4_ (and by extension that of AEX) is low overall, i.e., less than 6% on average, and that this novel measurement, AEX_4_, stratifies well between newly proposed categories of BD response (differentiating between negative, minimal, mild, moderate and marked responses by using FEV_1_ absolute *or* percent changes by the following thresholds: ≤ 0 mL/≤ 0%, ≤ 90 mL/≤ 9%, ≤ 160 mL/≤ 16%, ≤ 260 mL/≤ 26%, and ≥ 260 mL/≥ 26%, respectively). These observations suggest that AEX_4_ (AEX) is a useful measure for stratifying dysfunction in obstructive lung disease, and invite further analysis of AEX for evaluating clinical status and indications for using bronchodilator agents or disease-modifying, anti-inflammatory therapies.

The 2005 ATS/ERS guidelines define a significant BD response as an absolute 200 mL and a 12% change in either FEV_1_ or FVC [12]. However, establishing the ideal definition of a significant BD response is not an easy task and not without significant limitations [4–11, 18, 23, 24]. This is likely due to several factors: (1) BD responsiveness in its current form is a conservative dichotomous nominal parameter that does not capture very well clinically relevant reversibility of thoracic overdistension, air hyperinflation or gas trapping [8, 10, 25], (2) Conventional criteria fail to unequivocally differentiate between disease categories such as asthma and COPD [26], (3) BD non-responsiveness is likely not an optimal, defining criterion for Asthma-COPD overlap syndrome [27], (4) Standard BD response criteria may constitute too blunt or too insensitive a tool, especially for extreme lung function values [10], and (5) A standard ATS/ERS BD response does not correlate well with clinical response to bronchodilators, disease control or traditional functional assessments [26, 28, 29].

The recent reassessment of BD responsiveness criteria and the proposal of BD response strata by Hansen et al. was correlated with radiologic measurements, exercise performance, dyspnea scores, obstructive lung disease exacerbation frequency, and quality of life [13]. While the observations and definitions proposed by these authors are yet to be validated in other populations and assessed against patient centric, relevant outcomes, they likely represent a significant practical advance for clinicians, e.g., in helping guide use of bronchodilator agents, etc.

In the current point-of-care PFT data set, we found that 24% of the subjects tested demonstrated a standard BD response by either FEV_1_ or FVC criteria. Notably, this prevalence may be overestimated in that post-BD testing was only performed when the clinician suspected obstruction or airflow limitation, and ordered pre- and post-BD spirometry. Perhaps not unexpectedly, given the reliance on only FEV_1_ and the less stringent criteria (OR vs. AND operator) when using the new BD response criteria, only 23% of the group had a negative response (absolute or % change of ≤ 0 mL or 0%, respectively). The term ‘negative’ BD response may be a misnomer, as it does not exclude altogether the presence of airflow limitation: a global FEV_1_ decrement after albuterol administration may be due to a larger closing volume in certain areas of the lungs despite an increase in ventilation due to bronchodilatation in others, or due to progressive hyperinflation and gas trapping induced by repeated forced exhalation maneuvers. As such, this specific category may require further testing for identifying specific propensities to dynamically obstruct airflow. While the largest proportion of patients with a BD response was in the minimal category (45%), only 3.4% of them had a conventional BD response, which may get us closer to what the ‘reference’ group should be. Further, 45.5% of the tests deemed to have a mild BD response by the new criteria were found to have conventional positive BD reversibility. Clearly, these associations between conventional and novel BD responsiveness criteria must be re-assessed in independent, hypothesis-testing populations.

When juxtaposing the FEV_1_ vs AEX_4_ reversibility to inhaled BD, the topic of airway-parenchymal interaction becomes highly relevant, as lung volumes and degrees of hyperinflation influence not only the airway resistance, but also the bronchial responsiveness to bronchodilator or bronchoconstrictive agents [30–34]. As such, the use of only one parameter (e.g., FEV_1_ or airway resistance) in defining BD response has one inherent limitation, i.e., that the influence of FVC or other lung volumes is not taken into consideration. Indeed, bronchial responsiveness is highly influenced by the size of the end-expiratory lung volume, TLC, FRC or FVC [30–34]. While Hansen et al. [13] do propose to resort to only one parameter, this drawback is possibly minimized by a multi-layered approach, which may permit better endo-phenotypic characterizations. In our case, the AEX as a physiological measurement does not overlook the effects of lung volumes, as it is influenced by both FVC size and by any flow–volume curve ‘scooping’ or ‘shrinking’. This may in fact explain its low pre- to post-BD variability in comparison to that of FEF_75_, FEF_50_ or other ‘distal’ flows.

In previous work, we showed that square root (sqrt) AEX compared favorably with traditional PFT measurements for diagnosis and severity characterization of respiratory impairment. The sqrt AEX correlated also well with several lung volumes and capacities that characterize the degree of airway hyperinflation such as IC, IC/TLC and Residual Volume/TLC ratios. Further, several other evaluations of AEX and related concepts have been published before, mostly in children or in assessment of bronchoprovocation responses, and suggested that area under the flow–volume curve or similar constructs may be useful in special populations [35–39]. Due to these relationships, we posit that sqrt AEX offers the promise to become a good predictor of clinical symptoms and to lessen the need for complex lung volume testing [14, 15]. We are currently investigating intrinsic variation of various AEX parameters (inter-trial variance, both pre-and post-bronchodilator use), as well its utility in various spirometric patterns and conditions, for example the use of AEX variability in small airway disease.

The strengths of this study are: (1) the large size of the PFT data set, which represents a broad population, with a wide range of diseases; (2) the use of lung volume testing by other methods such as body plethysmography, helium dilution and DLCO in a large proportion of subjects tested, which allows establishing the ‘ground truth’, and (3) introduction of a global spirometric measurement, which incorporates effects of both airway resistance (flows) and volumes (FVC).

At the same time, several limitations of the study warrant discussion, including that: (1) all data come from a single center, with significant under-representation of women, potentially limiting generalizability, (2) lung volume and BD testing was performed at clinicians’ discretion, reflecting a potential clinical bias, and (3) details were lacking regarding participants’ underlying diagnoses and symptoms, the indication for pulmonary function testing, smoking status, and long-term outcomes, thereby limiting understanding of the clinical correlates of these findings.

## Conclusion

This study analyzed the variability of an approximated Area under the Expiratory flow–volume curve (AEX_4_) based on flows at peak expiration (PEF) and at predetermined volumes (FEF_25_, FEF_50_ and FEF_75_) for characterizing airway responsiveness, and using a newly proposed framework of bronchodilator responsiveness. As a functional parameter, AEX_4_ performs well as a surrogate marker of AEX, offering promise to help stratifying airway response patterns to inhaled bronchodilator agents and to better define clinical phenotypes and lung disease endotypes. Further studies are needed to examine the relationship of AEX_4_ to clinical symptoms, therapeutic impact and other patient centric outcomes, as well as best discriminating strata of AEX_4_.

## Abbreviations

AEX: Area under expiratory flow–volume curve
AEX_k_: AEX approximated based on k flows
ATS: American Thoracic Society
BD: Bronchodilator
BMI: Body Mass Index
CI: Confidence interval
COPD: Chronic obstructive pulmonary disease
DLCO: Diffusing lung capacity for CO
ERS: European respiratory society
FEF_xy_: Forced expiratory flow at xy% of FVC
FEF_25-75_: Forced expiratory flow between 25 and 75% of FVC
FEV_1_: Forced expiratory volume in 1 s
FET: Forced expiratory time
FET_PEF_: FET at PEF
FEV_PEF_: Forced expiratory volume at PEF
FEV_k_: Forced expiratory volume at k second of expiration
FIV_1_: Forced inspiratory volume in 1 s
FIVC: Forced inspiratory vital capacity
FRC: Functional residual capacity
FVC: Forced vital capacity
GLI: Global lung initiative
HSD: Honestly significant difference
IC: Inspiratory capacity
IQR: Interquartile Range
LLN: Lower limit of normal
PEF: Peak expiratory flow
PFT: Pulmonary function testing
TLC: Total lung capacity
SD: Standard deviation
Sqrt: Square root transformation (^^0.5^)
